# Geographical variations and potential low-value neuroimaging examinations in Norway from 2013 to 2022

**DOI:** 10.1007/s43999-025-00065-1

**Published:** 2025-05-21

**Authors:** Ingrid Øfsti Brandsæter, Jan Porthun, Eivind Richter Andersen, Bjørn Morten Hofmann, Elin Kjelle

**Affiliations:** 1 Department of Health Sciences Gjøvik at the Norwegian University of Science and Technology (NTNU) Gjøvik, PB 191, Gjøvik, 2802 Norway; 2 Centre for Medical Ethics at the University of Oslo, Blindern, Oslo, PB Norway

**Keywords:** Geographical variation, Neuroimaging, Diagnostic imaging, Low-value imaging

## Abstract

**Purpose:**

To investigate the variations in the use of neuroimaging over time and across geographical regions and to investigate the use of two potential low-value neuroimaging examinations for all imaging in an entire country (Norway), including both inpatient and outpatient services from 2013 to 2022.

**Method and methods:**

Data on neuroimaging for outpatients was collected from the Norwegian Health Economics Administration, and inpatient data were collected from individual Hospital Trusts (HT) in Norway. The data were analysed using descriptive statistics.

**Results:**

On average, 413,303 (786 per 10,000 inhabitants) neuroimaging examinations were performed annually in Norway. Overall, the use increased by 16% during the study period. Substantial geographical variations were found both in general and for the two potential low-value neuroimaging examinations; Brain Magnetic Resonance Imaging (MRI) and Head Computed Tomography (CT). For general neuroimaging, the HT with the highest use performed twice as many examinations as the HT with the lowest use per inhabitant. For the potential low-value neuroimaging examinations, the HTs with the highest use performed two and three times as many examinations as the HTs with the lowest use per inhabitant.

**Conclusion:**

There was temporal and geographical variation in the general use of neuroimaging and the use of the two potential low-value examinations, Brain MRI and Head CT. In Norway, the estimated annual cost of low-value neuroimaging examinations is about EUR 4.0 million. Reducing the use of low-value imaging would free up resources for examinations of high value.

**Supplementary Information:**

The online version contains supplementary material available at 10.1007/s43999-025-00065-1.

## Introduction

The use of diagnostic imaging has been increasing across the world for many decades [1]. Concerns about overutilisation and unnecessary use of diagnostic imaging are rising. Excessive imaging may indicate low-value imaging [2], which is defined as “an intervention where evidence suggests it confers no or very little benefit on patients, or risk of harm exceeds likely benefit, or, more broadly, the added costs of the intervention do not provide proportional added benefits” [3]. Between 20 and 50% of imaging worldwide is estimated to be low-value imaging [2, 4].

In neuroimaging, certain examinations have been identified to be of potentially low value. Examples are the use of Head Computed Tomography (CT) for minor head injury or headache, or Brain Magnetic Resonance Imaging (MRI)/Head CT for patients with migraine or uncomplicated headache [5, 6]. The international Choosing Wisely Initiative aims to reduce the use of such low-value care [7] and the campaign has been implemented in several countries, including Norway. In the Norwegian version, two neuroimaging examinations are included, and these are presented in Table 1 [8].

**Table 1:** The two Choosing Wisely Initiative recommendations for neuroimaging focused in the present study

Recommendation	Examples of red flags
Avoid diagnostic imaging (Brain MRI/Head CT) of the head for an uncomplicated headache, unless any red flags are present	Rapidly increasing frequency and severity of headache or lack of coordination
Avoid Head CT of adults after minor head trauma without any red flags	Glasgow Coma Scale less than 13, or less than 15 at 2 h post-injury, or obvious fracture of the skull

Low-value imaging may be reflected in geographical variation in imaging utilisation, as this is given by local habits and culture. Earlier research has documented geographical variation in diagnostic imaging in the USA [9–11], Europe [12–15], Australia [16], and in outpatient examinations in Norway [17–20].

Unwarranted geographical and temporal variations can indicate over- and underutilisation, which is a threat to the quality, safety and efficiency of care. Especially examinations identified as potential low-value imaging are one reason for overutilisation. Such variations break with the principle of equal access to care and justice. Moreover, unnecessary (low-value) examinations may generate unnecessary wait times and shortfalls, as there are long wait times for CT and MRI examinations in Norway [21]. Further, monitoring of geographical and temporal variations is also relevant for quality assurance in imaging, as this may indicate potential areas for improvement.

A large element of neuroimaging is performed on inpatients, and data are scattered across a range of systems, generating a knowledge gap for geographical and temporal variations for entire nations. Additionally, more information is needed about the use of potential low-value neuroimaging. Accordingly, this study aims to investigate the variations in the use of neurological imaging in Norway. This includes variation over time and across geographical regions, for all neuroimaging, both inpatient and outpatient services in Norway, from 2013 to 2022. Moreover, the study investigates geographical variations in the use of two well-documented potential low-value neuroimaging examinations: Brain MRI and Head CT. The objectives were:

1. How did neuroimaging in general vary across health regions in Norway and over time from 2013 to 2022?
2. How did two potentially low-value neuroimaging examinations vary across health regions in Norway and over time from 2013 to 2022?

## Material and methods

### Setting

Norway consists of urban, rural and remote areas. This means that some inhabitants face long travel distances to reach hospitals and imaging centres. Nonetheless, equal access to healthcare services is one of the key principles for Norwegian health services, regardless of where people live [22]. Norway is one of the European countries with the highest coverage of physicians and other health care services workers per inhabitant [22, 23]. Additionally, the Norwegian population is considered homogenous in regard og morbidity and the need for health care services, compared to other European countries [23, 24].

Diagnostic imaging is part of the specialist healthcare. In addition, non-profit hospitals and private imaging centres have contracts with healthcare authorities to conduct imaging on public terms [22]. There is a small co-pay amount for most of the outpatient care, including imaging. Patients can, however, get faster access to imaging by paying out-of-pocket or through health insurance. Around 10% of the population have private health insurance. For inpatient imaging, there is no co-pay fee [22].

Outpatient examinations are funded through reimbursement rates from the Norwegian Health Economics Administration (Helfo). Therefore, the hospitals report information on outpatient examinations to Helfo each year. Hence, Helfo has a register of all outpatient examinations conducted in hospitals and imaging centres in Norway.

**Fig. 1:**
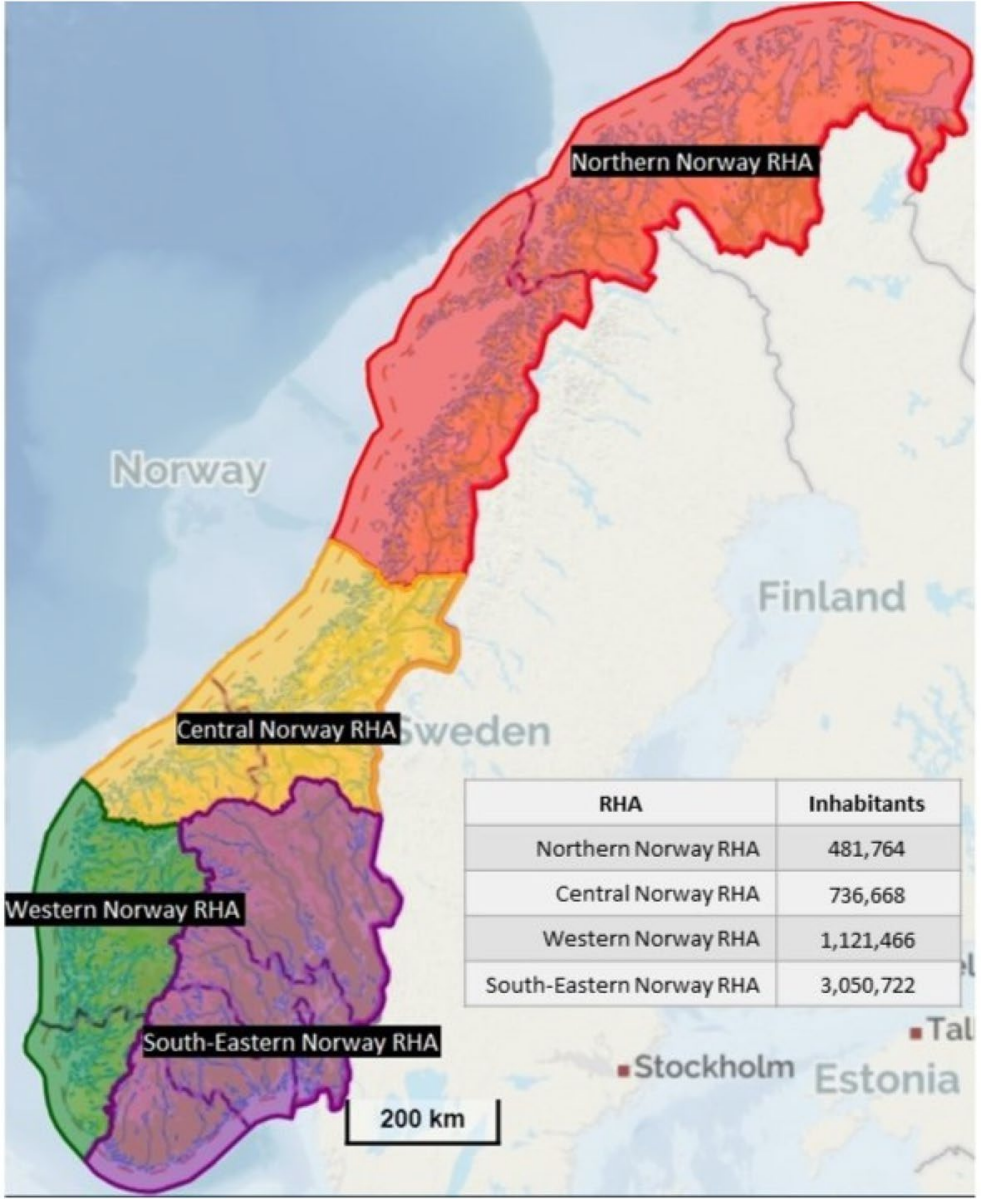
Overview of the four RHAs in Norway and the catchment areas’ population numbers 01.01.2021, Regional Health Authority (RHA)[26]

There are four Regional Health Authorities (RHAs) in Norway, which are responsible for a total of 19 Hospital Trusts (HTs) [25]. Figure 1 shows the four RHAs and the number of inhabitants. The capital of Norway, Oslo, is in Region South-East, and this RHA has a larger population than the other RHAs (56% of the total population in 2022). In addition, this RHA also hosts two national hospitals, i.e. the national hospital (Rikshospitalet – the University Hospital) and the national cancer hospital (Radiumhospitalet).

### Neuroimaging

To identify neuroimaging, the Norwegian Classification of Radiological Procedures (NCRP) codes [27] were used. In total, 65 NCRP codes relevant to neuroimaging were identified (supplementary file 1). These codes include procedures conducted using the modalities CT, MRI, Conventional Radiography (CR), Nuclear Medicine (NM) and Ultrasound (US). As the available data do not give access to clinical information, we were not able to assess whether patients underwent examinations for neurological issues. Thus, all relevant codes for neuroimaging were included.

For research question 2, the present study focuses on two potential low-value examinations presented in the introduction (Table 1): Brain MRI and Head CT. As we do not have the patient’s clinical information, we cannot know whether a specific examination is of low value or not, thus Brain MRI and Head CT are presented as “potential” low-value imaging in the present study. However, the levels of low-value imaging for these examinations are documented in the international literature [5, 6, 28–31].

A new set of NCRP codes was implemented in 2016. The examinations from 2013 to 2015 used the old codes, and these codes were therefore converted to the new codes in the present study.

### Data collection

Collected data included patients’ age and sex, NCRP code, imaging modality, hospital/imaging centre, and status as inpatient or outpatient.

Data on outpatients were collected from Helfo, and data on inpatients were collected from a selection of 15 HTs and non-profit hospitals. A selection was based on the HTs and the hospital’s geographical location and size.

### Statistical analysis

In total 11 hospitals missed data for the whole study period or part of the study period. Extrapolations were done to account for the inpatient data from the HTs and nonprofit hospitals (34.7% of inpatient examinations). All RHAs were represented in the collected inpatient data, including all inpatient data from one RHA. Therefore, the in- and outpatient distribution per inhabitant could be calculated. An extrapolation for the missing hospitals could be made based on the hospital's outpatient rate and the respective hospital’s catchment area inhabitant number and population distribution.

The geographical regions used in the analysis were based on the catchment areas for RHAs and HTs in Norway. The regions include the private imaging centres located in the respective HT and RHA’s catchment areas. Not all HTs had a private imaging centre located in the catchment area. Norwegian population data for each year was provided by Statistics Norway [26].

SPSS Statistics version 28 (IBM Corp.) and Microsoft Excel version 2305 were used for statistical analysis.

## Results

An average of 413,303 neuroimaging examinations were performed in Norway annually from 2013 to 2022, corresponding to 786 examinations per 10,000 inhabitants yearly. More females than males underwent neuroimaging (on average 54% and 46%). In total, the most used neuroimaging examinations in the study period were Brain MRI (35% of all neuroimaging) and Head CT (31% of all neuroimaging) followed by MRI, CR and CT of the cervical spine (10%, 4% and 4% of all neuroimaging).

**Fig. 2:**
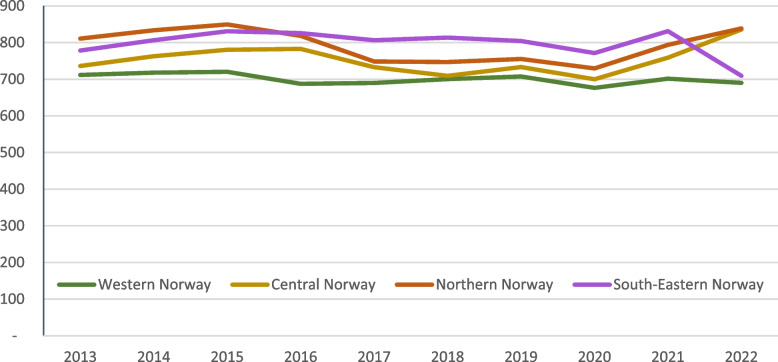
Temporal variations from 2013 to 2022 in the use of imaging modalities for neuroimaging in four regions in Norway per 10,000 inhabitants

The total number of neuroimaging examinations in Norway increased from 762 to 820 examinations per 10,000 inhabitants from 2013 to 2022, which corresponds with an overall increase of 16% from 2013 to 2022 in neuroimaging examinations. The number of images per 10,000 inhabitants varied over the last years with the lowest in 2020, the highest in 2022 and the second highest in 2015. Figure 2 shows the temporal variations in the four regions in Norway. Western Norway had the most stable trend, while the other three regions varied throughout the period.

**Fig. 3:**
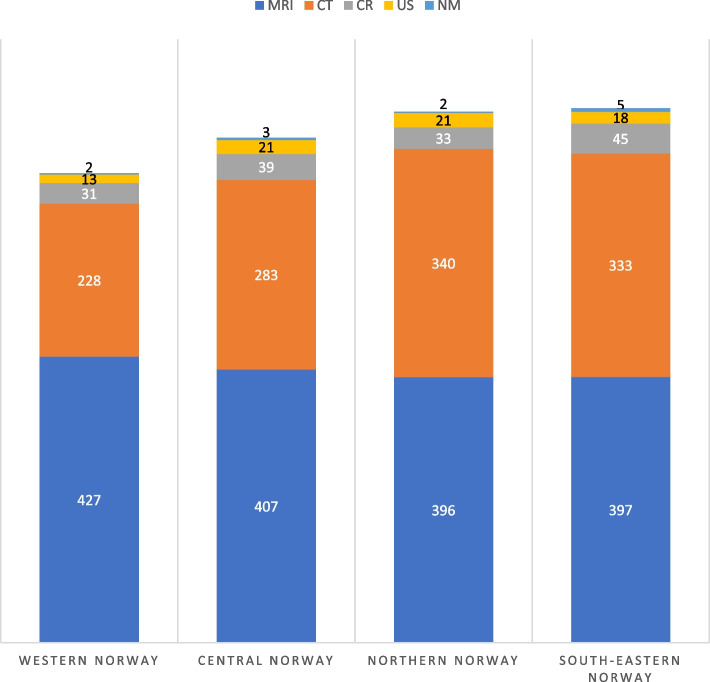
Geographical variation in the use of imaging modalities for neuroimaging in four regions in Norway per 10,000 inhabitants. Numbers are presented as the annual average from 2013 to 2022. MRI: Magnetic Resonance Imaging, CR: Conventional Radiology, CT: Computer Tomography, US: Ultrasound, NM: Nuclear Medicine

Overall, South-Eastern Norway had the highest relative use of neuroimaging, followed by Northern Norway RHA (1% lower relative use). Western Norway had the overall lowest relative use of neuroimaging. However, Western Norway had the highest relative use of MRI, 8% higher than Northern Norway which had the lowest relative use. Northern Norway had the highest relative use of CT, 49% higher than Western Norway. South-Eastern Norway had the highest use of CR and NM compared to the other regions. The use of US was lower in Western Norway than in the other regions. Details are shown in Fig. 3.

**Fig. 4:**
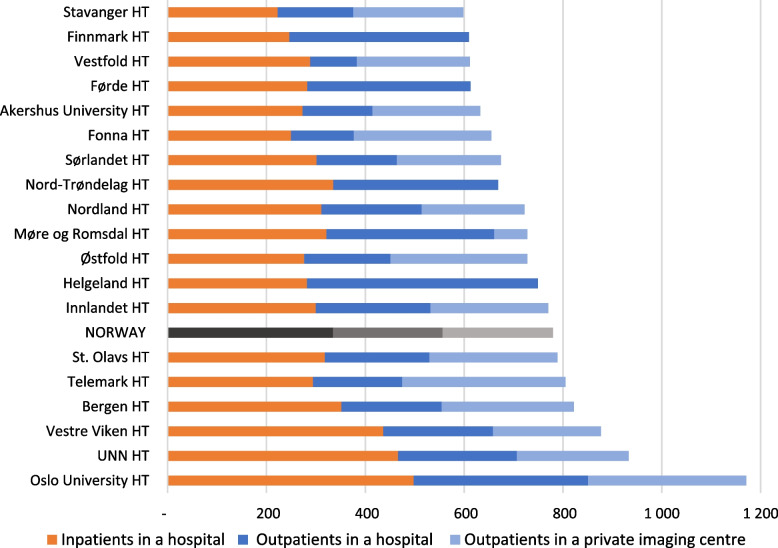
Variation in inpatient and outpatient neuroimaging between the 19 HTs catchment areas and the mean (NORWAY) performed in hospitals and private imaging centres. The HTs define the geographical regions and include all hospitals and imaging centres in the respective HT’s catchment area. Annual average for 2013–2022 in Norway, per 10,000 inhabitants. HT: Hospital Trust, UNN: University Hospital of North Norway

On average, 43% of the neuroimaging examinations in Norway were inpatients, while 50% of the outpatient examinations took place in private imaging centres. This varied between the HTs, as there are no private imaging centres within some HT’s catchment areas. Oslo University HT had the highest relative total use of neuroimaging with 98% higher use than Stavanger HT which had the lowest relative use (Fig. 4).

### Low-value neuroimaging

The two potential low-value neuroimaging examinations, Brain MRI and Head CT accounted for 66% (*n* = 2,741,204) of all neuroimaging examinations conducted between 2013 and 2022. Of these, 53% (*n* = 1,452,104) were Brain MRIs.

**Fig. 5:**
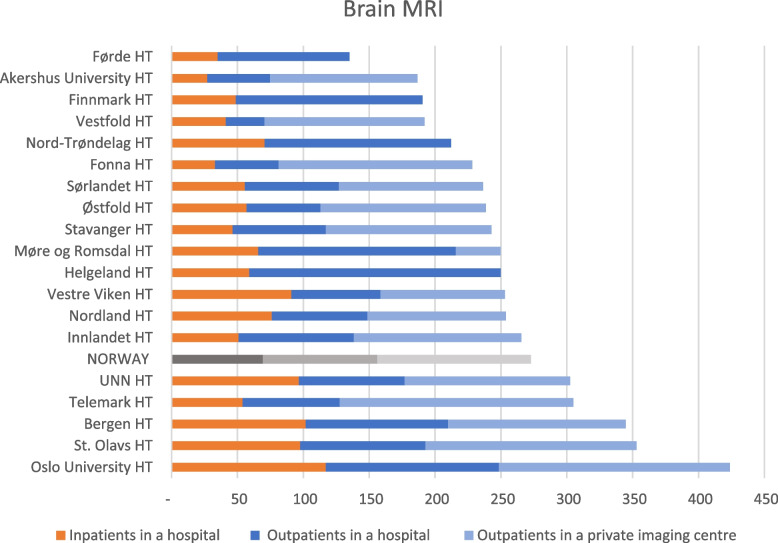
Variation in inpatient and outpatient Brain MRI between the 19 HTs catchment areas and the mean (NORWAY) performed in hospitals and private imaging centres. The HTs define the geographical regions and include all hospitals and imaging centres in the respective HT’s catchment area. Annual average for 2013–2022 in Norway, per 10,000 inhabitants. HT: Hospital Trust, UNN: University Hospital of North Norway

The use of Brain MRI increased by 22.8% from 2013 to 2022. The catchment area of Oslo University HT had the highest use per inhabitant, with three times as many examinations as Førde HT, which had the lowest use per inhabitant. On average, 43% of the Brain MRIs were conducted in private imaging centres and 25% were of inpatients in hospitals (Fig. 5).

**Fig. 6:**
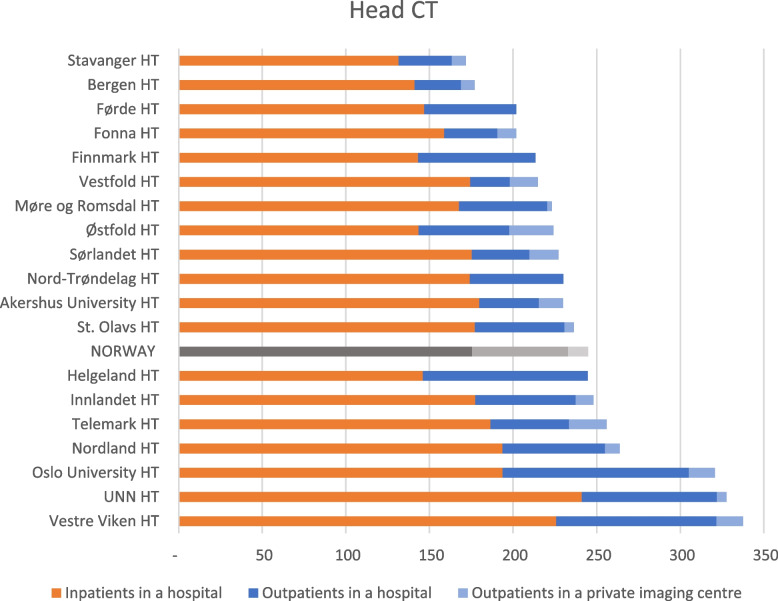
Variation in inpatient and outpatient Head CT between the 19 HTs catchment areas and the mean (NORWAY) performed in hospitals and private imaging centres. The HTs define the geographical regions and include all hospitals and imaging centres in the respective HT’s catchment area. Annual average for 2013–2022 in Norway, per 10,000 inhabitants. HT: Hospital Trust, UNN: University Hospital of North Norway

The number of Head CTs performed in Norway increased by 10.3% from 2013 to 2022. Vestre Viken HT had the highest relative use, twice as high as Stavanger HT, which had the lowest relative use. On average, 5% of the examinations were conducted at private imaging centres, and 72% of the Head CTs were performed as inpatient examinations in hospitals (Fig. 6).

The total combined number of Brain MRI and Head CT in Norway increased by 16.5% during the study period, corresponding to a change from 512 to 555 examinations per 10,000 inhabitants from 2013 to 2022. And 2022 was the year with the highest use per 10,000 inhabitants, while 2020 had the lowest use.

**Fig. 7:**
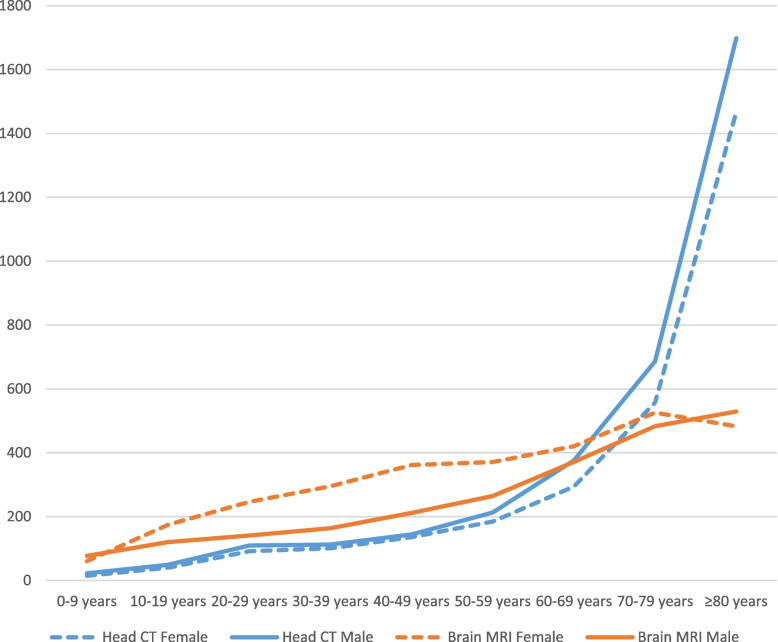
Variation between the number of examinations of females and males in different age groups, for Head CT and Brain MRI, respectively. Annual average for 2013–2022 in Norway, per 10,000 inhabitants. CT: Computer Tomography, MRI: Magnetic Resonance Imaging

There was a greater difference between males and females for Brain MRI than for Head CT. For Brain MRI, 57% of the patients were female, but for Head CT, 49% of the patients were female. For Brain MRI, the greatest difference between males and females was observed for the 40–49 age group. Conversely, concerning Head CTs, the most noteworthy distinction between males and females occurred in the ≥ 80 age group. Both examinations saw a small increase in use from age groups 0–9 to 50–59. For these age groups, Brain MRI was used more than Head CT. However, from 70 years and older, the use of Head CT saw a large increase per 10,000 inhabitants. The distribution between females and males for the different age groups is shown in Fig. 7.

## Discussion

This study demonstrated substantial temporal and geographical variations in the use of neuroimaging between the catchment areas of the 19 HTs, and between and within the four RHAs in Norway. The study does not measure the proportion of low-value imaging, but geographical variations can indicate that low-value imaging is conducted in Norway. However, the international literature indicates the level of low-value imaging for these examinations [5, 6, 28–31]. The catchment area of the HT with the highest relative use performed twice as many examinations as the HT with the lowest relative use. The number of neuroimaging examinations increased by 16% from 2013 to 2022. There are also geographical variations in the use of two low-value neuroimaging examinations: Head CT and Brain MR. The HTs with the highest relative use of these potential low-value examinations conducted two and three times as many examinations as the HTs with the lowest relative use.

The result in the present study is in line with previous research that also found geographical variations in outpatient imaging in Norway [20, 32, 33]. In this study, one HT conducted three times as many Brain MRIs as another HT, this diverges from another Norwegian study that found small variations in the use of Brain MRI [17]. However, this study adds to the previous literature by including inpatient data, hence, providing a more accurate picture of imaging utilization in Norway.

Oslo HT had the highest use of neuroimaging and Brain MRI and was among the top three in use of Head CT. Two specialist hospitals that cover the entire population are located in Oslo University HT and the data in this study are based on where the examination was conducted, not the patient’s residence. These two hospitals receive patients from the whole country and specialist migration may therefore be a reason for the high use by the Oslo University HT.

One reason for the geographical variations can be access to healthcare services and equipment availability. Studies have demonstrated that easy access to imaging is a reason for geographical variation and low-value imaging [34–36]. Private imaging centres conduct imaging on behalf of the RHAs. The imaging centres are mainly located in big and small cities in Norway. Only four regions do not have a private imaging centre within their catchment area. This can explain why Førde has the lowest use of Brain MRI (Fig. 5). However, the other three are not at the bottom of the list of Bran MRI use, nor the list of Head CT or neuroimaging in general. Further, studies have shown that the Norwegian population living in rural areas with high travel distances to health care are willing to travel for diagnostic imaging [37, 38]. Most patients choose a hospital in their region to receive healthcare [39] even though patients have the right to choose the place of healthcare service, regardless of geographical location [40].

The present study does not include examinations paid out-of-pocket or paid through private health insurance. Between 10 and 14% of the population has private health insurance [22, 37]. The lack of examinations paid through private health insurance data can underestimate the amount of imaging in the areas with private imaging centres.

The use of Brain MRI increased by 22.8% and the use of Head CT increased by 10.3% during the study period. The temporal use of these examinations varied within the HT’s catchment areas, and the level of variation differed between the HTs. There was a decrease in the use of these low-value neuroimaging in 2020 and this is also seen in another study [41], although the utilisation increased towards the end of 2020. The number of inhabitants in Norway increased by 8% from 2013 to 2022 [42].

Brain MRI and Head CT are the two most used neuroimaging in Norway, in accordance with earlier research [32, 33, 41]. These two examinations are well documented to be low-value for the aforementioned patient groups [5–7, 43]; for patients with minor head injuries, only 2.0–7.4% of Head CTs provide relative findings [28–30], and for patients with migraine, Brain MRI or Head CT are not even recommended in guidelines [31]. Also for children, low-value CT and MRI examinations of the head and brain were most frequently reported [5]. Further, one cannot know the amount of low-value Bran MRI and Head CT in Norway based on the present study because the indications for and outcomes of the examinations are unknown. Studies have demonstrated that between 20 and 50% of diagnostic imaging worldwide is of low value [2, 4]. A conservative estimate of 20% of low-value examinations would result in 28,703 Brain MRIs and 25,767 Head CTs annually in Norway. Based on cost estimates from a study that calculated average costs for these low-value examinations [44], the costs per Brain MRI and Head CT were EUR 82 and EUR 63, respectively. This means that a 20% reduction in Brain MRI and Head CT together would result in around EUR 4.0 million freed up for examinations of high value.

The results demonstrate different use of Head CT and Brain MRI for men and women, and for different age groups. This is in line with a previous study in Norway, which found that twice as many women as men, aged between 15 and 55, underwent a Brain MRI [33]. One explanation for this might be that headaches and migraines are more frequent for women than men [45]. The largest difference in Head CT between men and women was for age groups over 50 years old. This difference may be explained by different occurrences of disease for men and women. In Norway, for instance, a higher incidence of stroke is observed in men compared to women. These cases are routinely investigated by a Head CT scan [46].

### Strengths and limitations

One strength of the study is that the data includes both inpatients and outpatients. As data on inpatient examinations must be retrieved from each HT, which has different digital systems, we did not get data on all inpatient examinations (34.7% of inpatient data was extrapolated). However, the total number of collected inpatient and outpatient data covered 90.5% of the total dataset. The collected data were representative of the whole population, and we made robust extrapolations. Some US examinations are conducted in other hospital departments than radiology and are thus registered in other systems and not included in this study.

One limitation of the study is the potentially different uses of the NCRP codes [17]. Some of the codes are similar to each other and there are several types of combination codes, which may give inconsistent use. Hospitals and private centres may have different coding routines which can influence the results. Another limitation is that the data was not adjusted for age and sex due to the small differences between the regions’ catchment areas.

The data is not adjusted for sex and age, this could be an explanation for some of the geographical variations. The population living in rural areas is in general older compared to the population in the cities [47] and Fig. 7 shows that Brain MRI and Head CT are most used in patients over 70 years old. On the other hand, the biggest cities of Norway are located in the areas with the highest use of neuroimaging which, in general, has a younger population.

The system for NCRP codes changed in 2016. Although all codes were converted to the 2016 version, some codes were merged, and others divided, which might have affected the data. In addition, the hospitals and HTs might have different routines for the use of the NCRP codes, which could affect the results. In the overall results, however, all codes similar to each other are included, resulting in small variations.

Further research should explore the causes of geographical variations and aim to find the ‘correct’ level of diagnostic imaging utilisation. Further studies should also investigate whether the healthcare services are equitable for the Norwegian population.

## Conclusions

Both temporal and geographical variations were found in this study. The total number of neuroimaging examinations in Norway increased by 16% from 2013 to 2022, however the highest use of neuroimaging per capita was in 2022 and the lowest use was in 2020. In addition, variations were demonstrated for the use of the two potentially low-value examinations identified: Brain MRI and Head CT. Low-value examinations take up a lot of resources, and the cost of low-value neuroimaging examinations in Norway is about EUR 4 million per year, based on estimations from this paper. Reducing the use of low-value imaging would free up resources for examinations of high value.

## Supplementary Information

Supplementary Material 1.

## Data Availability

The datasets generated and analysed during the current study are not publicly available due to regulation by the REC. However, aggregated data are available from the corresponding author on reasonable request.

## References

[1] Brady A, Brink J, Slavotinek J (2020) Radiology and value-based health care. JAMA 324(13):1286–1287 32915190 10.1001/jama.2020.14930

[2] Hendee WR, Becker GJ, Borgstede JP, Bosma J, Casarella WJ, Erickson BA et al (2010) Addressing overutilization in medical imaging. Radiology 257(1):240–245 20736333 10.1148/radiol.10100063

[3] Scott IA, Duckett SJ (2015) In search of professional consensus in defining and reducing low-value care. Med J Aust 203(4):179–181 26268286 10.5694/mja14.01664

[4] Sheng AY, Castro A, Lewiss RE (2016) Awareness, Utilization, and Education of the ACR Appropriateness Criteria: A Review and Future Directions. J Am Coll Radiol 13(2):131–136 26499160 10.1016/j.jacr.2015.08.026

[5] Kjelle E, Andersen ER, Krokeide AM, Soril LJJ, van Bodegom-Vos L, Clement FM et al (2022) Characterizing and quantifying low-value diagnostic imaging internationally: a scoping review. BMC Med Imag 22(1):73 10.1186/s12880-022-00798-2 PMC9022417 35448987

[6] Levin DC, Rao VM (2017) Reducing inappropriate use of diagnostic imaging through the Choosing Wisely initiative. J Am Coll Radiol 14(9):1245–1252 28457815 10.1016/j.jacr.2017.03.012

[7] Choosing Wisely (2017) The Choosing Wisely lists [18.08.2022]. Available from: https://www.choosingwisely.org/getting-started/lists/

[8] Norsk radiologisk forening. Gjør kloke valg-Radiologi [English title: Choosing Wisely - radiology] [23.05.2023]. Available from: https://www.legeforeningen.no/foreningsledd/fagmed/norsk-radiologisk-forening/artikler/fag-og-utdanningsstoff-fra-noraforum/gjor-kloke-valg-radiologi/

[9] Arnold RW, Graham DA, Melvin PR, Taylor GA (2011) Variability in imaging utilization in US pediatric hospitals. Pediatr Radiol 41(7):867–74 21301826 10.1007/s00247-011-1998-2

[10] Couchman GR, Forjuoh SN, Reis MD, Bartels G, Lindzey D (2005) Variation in MRI/CT utilization among family physicians and general internists in a multi-specialty group practice. Med Sci Monit 11(3):19–25 15735575

[11] Parker L, Levin DC, Frangos A, Rao VM (2010) Geographic variation in the utilization of noninvasive diagnostic imaging: national medicare data, 1998–2007. AJR Am J Roentgenol 194(4):1034–1039 20308507 10.2214/AJR.09.3528

[12] Nuti S, Vainieri M (2012) Managing waiting times in diagnostic medical imaging. BMJ Open 2(6):e001255 10.1136/bmjopen-2012-001255 PMC3533122 23242480

[13] Dreisbach JG, Nicol ED, Roobottom CA, Padley S, Roditi G (2018) Challenges in delivering computed tomography coronary angiography as the first-line test for stable chest pain. Heart 104(11):921–927 29138258 10.1136/heartjnl-2017-311846 PMC5969350

[14] O’Sullivan JW, Stevens S, Oke J, Hobbs FDR, Salisbury C, Little P et al (2018) Practice variation in the use of tests in UK primary care: a retrospective analysis of 16 million tests performed over 3.3 million patient years in 2015/16. BMC Med 16(1):229 30567539 10.1186/s12916-018-1217-1 PMC6300913

[15] Pieri C, Bhuva A, Moralee R, Abiodun A, Gopalan D, Roditi GH et al (2021) Access to MRI for patients with cardiac pacemakers and implantable cardioverter defibrillators. Open Heart 8(1):e001598 10.1136/openhrt-2021-001598 PMC8149430 34031214

[16] Fonseca R, Otahal P, Wiggins N, Marwick TH (2015) Growth and geographical variation in the use of cardiac imaging in Australia. Intern Med J 45(11):1115–1127 26247783 10.1111/imj.12867

[17] Riksrevisjonen (2017) Riksrevisjonens undersøkelse av bruken av poliklinisk bildediagnostikk [English title: The Office of the Auditor General of Norway's investigation of the use of outpatient diagnostic imaging. Available from: https://www.riksrevisjonen.no/globalassets/rapporter/no-2016-2017/bildediagnostikk.pdf

[18] Espeland A, Natvig NL, Loge I, Engebretsen L, Ellingsen J (2007) Magnetic resonance imaging of the knee in Norway 2002–2004 (national survey): rapid increase, older patients, large geographic differences. BMC Health Serv Res 7:115 17659090 10.1186/1472-6963-7-115 PMC1959197

[19] Gransjøen AM, Lysdahl KB, Hofmann BM (2019) Geographical variations in the use of diagnostic imaging of musculoskeletal diseases in Norway. Acta Radiol 60(9):1153–1158 30417668 10.1177/0284185118812204

[20] Lysdahl KB, Borretzen I (2007) Geographical variation in radiological services: a nationwide survey. BMC Health Serv Res 7:21 17302970 10.1186/1472-6963-7-21 PMC1805434

[21] Hofmann B, Brandsaeter IØ, Kjelle E (2023) Variations in wait times for imaging services: a register-based study of self-reported wait times for specific examinations in Norway. BMC Health Serv Res 23(1):1287 37996873 10.1186/s12913-023-10284-2 PMC10666297

[22] Saunes IS (2020) The norwegian health care system. In: Tikkanen R, Osborn R, Mossialos E, Djordjevic A, Wharton G, editors. International Profiles of Health Care Systems p. 159–68

[23] NOU 2023: 4. Tid for handling — Personellet i en bærekraftig helse- og omsorgstjeneste. Departementenes sikkerhets- og serviceorganisasjon, Teknisk redaksjon, editors. [English title: NOU 2023: 4. Time for action — The personnel in a sustainable healthcare service. Norwegian Government Security and Service Organisation]. Available from: https://www.regjeringen.no/no/dokumenter/nou-2023-4/id2961552/

[24] Senter for klinisk dokumentasjon og evaluering (SKDE) (2015) Dagkirurgi i Norge 2011–2013, Utvalgte inngrep. Senter for klinisk dokumentasjon og evaluering, Tromsø [English title: Day Surgery in Norway 2011–2013, Selected Procedures. Centre for Clinical Documentation and Evaluation]

[25] Braut GS (2022) Regionalt helseforetak Store medisinske leksikon. Available from: http://sml.snl.no/regionalt_helseforetak

[26] Norway) SsS. 07459: Alders- og kjønnsfordeling i kommuner, fylker og hele landets befolkning (K) 1986 - 2023 2023 [06.06.2023]. Available from: https://www.ssb.no/statbank/table/07459/

[27] The Directorate of e-health (2023) Norsk klinisk prosedyrekodeverk (NKPK), Alle prosedyrekoder 2023 oppdatert 21.11.2022 (excel). Available from: https://www.ehelse.no/kodeverk-og-terminologi/Norsk-klinisk-prosedyrekodeverk-(NKPK)

[28] Ali AHA, Al-Ghamdi S, Karrar MH, Alajmi SA, Almutairi OS, Aldalbahi AM et al (2018) Is there a misuse of computed tomography in the diagnostic workup of headache? A retrospective record-based study in secondary health-care facility in Saudi Arabia. J Family Med Prim Care 7(2):357–361 10.4103/jfmpc.jfmpc_338_17 PMC6060924 30090777

[29] You JJ, Gladstone J, Symons S, Rotstein D, Laupacis A, Bell CM (2011) Patterns of care and outcomes after computed tomography scans for headache. AM J MED 124(1):58-63.e1 20961529 10.1016/j.amjmed.2010.08.010

[30] Campiglio L, Bianchi F, Cattalini C, Belvedere D, Rosci CE, Casellato CL et al (2017) Mild brain injury and anticoagulants. Neurol Clin Pract 7(4):296–305 29185534 10.1212/CPJ.0000000000000375 PMC5648198

[31] Peres MFP, Swerts DB, de Oliveira AB, Silva-Neto RP (2019) Migraine patients’ journey until a tertiary headache center: an observational study. J Headache Pain 20(1):88 31416424 10.1186/s10194-019-1039-3 PMC6734236

[32] Hofmann BM, Gransjøen AM (2022) Geographical variations in the use of outpatient diagnostic imaging in Norway 2019. Acta Radiol Open 11(2):20584601221074561 10.1177/20584601221074561 PMC8891857 35251700

[33] Senter for klinisk dokumentasjon og evaluering (SKDE). Helseatlas radiologi første del, MR [English title: Health atlas radiology first part, MRI] 2023 [31.08.2023]. Available from: https://apps.skde.no/helseatlas/v2/radiologi/

[34] Lysdahl KB, Hofmann BM (2009) What causes increasing and unnecessary use of radiological investigations? A survey of radiologists’ perceptions. BMC Health Serv Res 9:155 19723302 10.1186/1472-6963-9-155 PMC2749824

[35] Pagano E, Di Cuonzo D, Bona C, Baldi I, Gabriele P, Ricardi U et al (2007) Accessibility as a major determinant of radiotherapy underutilization: a population based study. Health Policy 80(3):483–491 16781002 10.1016/j.healthpol.2006.05.006

[36] Brandsæter IØ, Andersen ER, Hofmann BM, Kjelle E (2023) Drivers for low-value imaging: a qualitative study of stakeholders’ perspectives in Norway. BMC Health Serv Res 23(1):295 36978092 10.1186/s12913-023-09328-4 PMC10044073

[37] Andersen ER, Brandsæter I, Hofmann BM, Kjelle E (2023) The use of low-value imaging: the role of referral practice and access to imaging services in a representative area of Norway. Insights Imaging 14(1):29 36746848 10.1186/s13244-023-01375-z PMC9902580

[38] Mokienko A (2019) Effects of a reimbursement change and travel times on the delivery of private and public radiology services in Norway: a register-based longitudinal study of Norwegian claims data. Cost Eff Resour Alloc 17(1):22 31636513 10.1186/s12962-019-0190-7 PMC6796397

[39] Vrangbaek K, Østergren K, Birk HO, Winblad U (2007) Patient reactions to hospital choice in Norway, Denmark, and Sweden. Health Econ Policy Law 2(Pt 2):125–152 18634659 10.1017/S174413310700401X

[40] Ministry of Health and Care Services (1999) Lov om pasient- og brukerrettigheter (pasient- og brukerrettighetsloven) [English title: Act on patient and user rights (patient and user rights act)] [01.08.2024]. Available from: https://lovdata.no/pro/NL/lov/1999-07-02-63

[41] Hofmann B, Andersen ER, Kjelle E (2021) What can we learn from the SARS-COV-2 pandemic about the value of specific radiological examinations? BMC Health Serv Res 21(1):1158 34702243 10.1186/s12913-021-07190-w PMC8546787

[42] Statistics Norway. Tabellnr. 07459: Alders- og kjønnsfordeling i kommuner, fylker og hele landets befolkning (K) 1986 - 2023 [English title: Table no. 07459: Age and sex distribution in municipalities, counties and the country's entire population (K) 1986 - 2023] 2023 [06.06.2023]. Available from: https://www.ssb.no/statbank/table/07459/

[43] Gjør Kloke Valg: Norsk Radiologisk forening (Norwegian) [12.09.2023]. Available from: https://www.legeforeningen.no/foreningsledd/fagmed/norsk-radiologisk-forening/artikler/fag-og-utdanningsstoff-fra-noraforum/gjor-kloke-valg-radiologi/

[44] Tolstjakova J (2022) Kostnader knyttet til lavverdi-undersøkelser innen bildediagnostikk. Master Thesis. University of Oslo, Norway

[45] Hagen K, Stovner LJ, Zwart JA (2020) Time trends of major headache diagnoses and predictive factors. Data from three Nord-Trøndelag health surveys. J Headache Pain 21(1):24 32160857 10.1186/s10194-020-01095-5 PMC7066736

[46] Nasjonalt sekretariat for Norsk hjerneslagregister SOhH (2021) Hjerneslag i Norge 2021

[47] Christiansen STG, Folkehelseinstituttet. Befolkningen i Norge 2022 [28.03.2025]. Available from: https://www.scopus.com/search/form.uri?display=basic#basic

