# Supplementary material for: Geographical variations and potential low-value neuroimaging examinations in Norway from 2013 to 2022
Source: Res Health Serv Reg. 2025 May 21;4:6. doi: 10.1007/s43999-025-00065-1 (PMC12095830; doi:10.1007/s43999-025-00065-1)
Supplement: Supplementary file 1 — Supplementary Material 1. [file 43999_2025_65_MOESM1_ESM.pdf]

Supplementary File 1: NCRP codes relevant for neuroimaging examinations

NCRP code	Examination
**SAA0AA**	CR Head
**SAB0CA**	CR Cervical myelography
**SNA0AA**	CR Cervical spine
**SSH0AB**	CR Arteriography of arteries in head and throat
**SAA0AB**	CR Cerebral arteriography
**SAA0AC**	CR Cerebral venography
**SSH0AC**	CR Venography of veins in head and throat
**SAA0AD**	CT Head
**SAA0DE**	CT Head and CT arteriography of head
**SAA0DP**	CT Head and CT angiography of head
**SNA0AD**	CT Cervical spine
**SAA0AP**	CT Angiography of head
**SSH0AP**	CT Angiography of head and throat
**SAA0AF**	CT Venography of head
**SPH0AF**	CT Venography of throat
**SAA0AE**	CT Arteriography of head
**SPA0AE**	CT Arteriography of throat
**SSH0AE**	CT Arteriography of arteries in head and throat
**SAA0AG**	MRI Brain
**SNA0NG**	MRI Brain and part of spine
**SAA0GH**	MRI Brain and MRI Arteriography of head
**SAA0GQ**	MRI Brain and MRI angiography of head
**SNA0LG**	MRI Brain and total spine
**SNA0AG**	MRI Cervicale spine
**SSH0AG**	MRI Brain and throat
**SAA0AQ**	MRI Angiography of head
**SPA0AQ**	MRI Angiography of throat
**SSH0AQ**	MRI Angiography of head and throat
**SAA0AJ**	MRI Venography of head
**SPH0AJ**	MRI Venography of throat
**SSH0AJ**	MRI Venography of veins in head and throat
**SAA0AH**	MRI Arteriography of head
**SPA0AH**	MRI Arteriography of throat
**SSH0AH**	MRI Arteriography of arteries in head and throat
**SAA0AK**	US Head
**SAA0BK**	US Head with Doppler
**SPA0AK**	US Arteries in throat
**SPH0AK**	US Veins in throat
**SSH0AK**	US Head and throat
**SNA0AK**	US Spine
**TAA0AN**	NM rCBF

**TSY0GN**	NM Shunt quantification
**TAA0EN**	NM Shunt examination
**AA0AL**	PET/CT Brain
**AA0AM**	PET/MRI Brain
**TAA0JL**	PET/CT - 18F Flurpiridaz Brain
**TAA0HL**	PET/CT - 18F PSMA Brain
**TAA0WL**	PET/CT Brain, other radiopharmaceuticals
**TAA0EL**	PET/CT-18F DOPA Brain
**TAA0BL**	PET/CT-18F FACBC Brain
**TAA0AL**	PET/CT-18F FDG Brain
**TAA0CL**	PET/CT-18F flutemetamol Brain
**TAA0DL**	PET/CT-18F NaF Brain
**TAA0GL**	PET/CT-64Cu DotaX Brain
**TAA0FL**	PET/CT-68Ga DotaX Brain
**TAA0JM**	PET/MRI - 18F Flurpiridaz Brain
**TAA0HM**	PET/MRI - 18F PSMA Brain
**TAA0WM**	PET/MRI Brain, other radiopharmaceuticals
**TAA0EM**	PET/MRI-18F DOPA Brain
**TAA0BM**	PET/MRI-18F FACBC Brain
**TAA0AM**	PET/MRI-18F FDG Brain
**TAA0CM**	PET/MRI-18F flutemetamol Brain
**TAA0DM**	PET/MRI-18F NaF Brain
**TAA0GM**	PET/MRI-64Cu DotaX Brain
**TAA0FM**	PET/MRI-68Ga DotaX Brain
